# Effect of Varying Normal Stiffness on Soft Rock Joints under Cyclic Shear Loads

**DOI:** 10.3390/ma16124272

**Published:** 2023-06-08

**Authors:** S. M. Mahdi Niktabar, K. Seshagiri Rao, Amit Kumar Shrivastava, Jiří Ščučka

**Affiliations:** 1 Department of Laboratory Research on Geomaterials, Institute of Geonics of the Czech Academy of Sciences, Studentska 1768/9, 708 00 Ostrava, Czech Republic; jiri.scucka@ugn.cas.cz; 2 Department of Civil Engineering, Indian Institute of Technology Delhi, Delhi 110016, India; raoks.iitd@gmail.com; 3 Department of Civil Engineering, Delhi Technological University, Delhi 110042, India; aksrivastava@dce.ac.in

**Keywords:** soft rock joints, cyclic shear loads, rock joint roughness, normal stiffness, regular and irregular joints

## Abstract

The evaluation of changes in shear resistance on soft (or weathered) rock joints under cyclic shear loads with constant normal load (CNL) and constant normal stiffness (CNS) significantly contributes to increasing the safety and stability of rock slopes and underground structures. In this study, a series of cyclic shear tests were conducted on simulated soft rock joints with regular (15°-15°, 30°-30°) and irregular (15°-30°) asperities under different normal stiffnesses (*k_n_*). The results indicated that the first peak shear stress increases with the increase in *k_n_* up to the normal stiffness of the joints (*k_nj_*). Beyond *k_nj_*, no significant change was observed in the peak shear stress. The difference in peak shear stress between regular (30°-30°) and irregular joints (15°-30°) increases as *k_n_* increases. The minimum difference of peak shear stress between regular and irregular joints was observed (8.2%) under CNL and the maximum difference was found (64.3%) on *k_nj_* under CNS. The difference in peak shear stress between the first and subsequent cycles significantly increases as both the joint roughness and *k_n_* increases. A new shear strength model is developed to predict peak shear stress of the joints for different *k_n_* and asperity angles under cyclic shear loads.

## 1. Introduction

**Figure 1:**
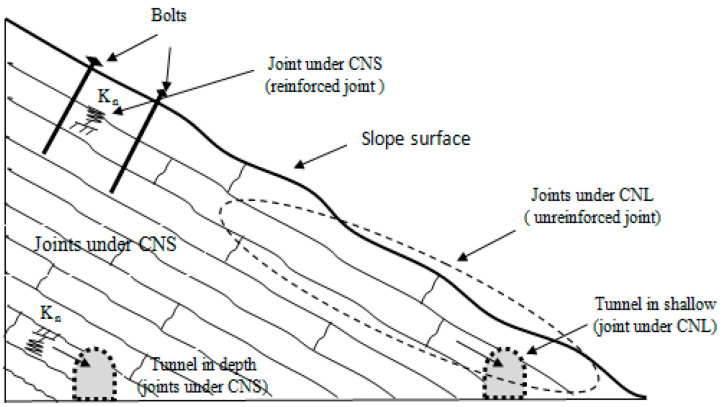
Sketch of rock mass under CNL and CNS boundary conditions.

Joints are exposed to different boundary conditions: constant normal load (CNL) and constant normal stiffness (CNS), as shown in Figure 1. Under CNL, the normal stress is constant and joints are free to dilate, whereas under CNS, the normal stress is not constant and joint dilation is partially or fully restricted by the surrounding rock mass. Normal stress changes depending on the normal stiffness (*k_n_*), which is related to the depth (Jiang et al., 2009 [1]) or stiffness of a bolt in a reinforced joint and the roughness of the shearing planes.

If dynamic loads (e.g., due to blasting or seismic activities) propagate in the rock mass, they can generate cyclic shear loads (vibration) along the joints and thus significantly increase the risk of rock mass failure. An increase in the number of shear cycles under dynamic loads leads to a reduction in the shear resistance of joints. Therefore, the evaluation of changes in shear resistance under cyclic loads significantly contributes to increasing the safety and stability of underground structures, rock slopes, foundations, and rock socketed piles.

Numerous studies have been published on the shear behaviour of rock joints. Shear strength models have been presented based on monotonic shear loads and asperity angle (or joint roughness coefficient (JRC)), under CNL (Paton, 1966 [2]; Ladany et al. [3], 1970; Jaeger, 1971 [4]; Barton 1973 [5]; Barton and Choubey, 1977 [6]; Amadei and Saeb, 1990 [7]; Kulatilake et al., 1995 [8]; Seidel and Haberfield, 1995 [9]; Grasselli et al., 2002 [10]; Asadollahi and Tonon, 2010 [11]) and under CNS (Heuze, 1979 [12]; Johnston and Lam, 1989 [13]; Saeb and Amadei, 1992 [14]; Skinas et al., 1990 [15]; Indraratna et al., 1998 [16]; Haque, 1999 [17]; Indraratna and Haque, 2000 [18]; Shrivastava and Rao, 2018 [19]). However, only a few studies have been conducted on jointed rocks under both CNS and cyclic shear loads.

In the past, most studies on cyclic shear loads were carried out under CNL. A series of cyclic shear tests was performed on rock joints (granite, limestone, and dolomite) under the CNL condition (Hutson and Dowding, 1990 [20]; Huang et al., 1993 [21]). The reports indicated that joint shear resistance and dilation at the first cycle were more pronounced as compared with subsequent cycles. In addition, shear resistance was decreased because of increasing asperity degradation during cyclic shear loads. However, the joint was insensitive in the case of granite (due to the high strength of the rock). Similar studies have been conducted on rock joints, and constitutive models (Plesha, 1987 [22]; Qiu et al., 1993 [23], Jing et al., 1993 [24]) have been proposed to predict the shear behaviour of rock joints under cyclic loading. The cyclic shear behaviour of saw cut and tensile splitting joints in two types of rocks (granite and marble) was investigated by Lee et al. (2001) [25] under CNL. Frictional resistance increased gradually with an increase in the number of cycles in case of a saw cut joint in granite, whereas no change was observed for marble. They also proposed an elasto-plastic model for degradation of second-order asperities. Jafari et al. (2003) [26] conducted cyclic shear tests on joints (triangular asperities and real joint replicas) at three different levels of normal stress under CNL and developed mathematical models for shear strength. It was observed that the shear sliding mechanism was significant at low normal stress and that the second-order asperities were not effective during shear cycles. However, in another report for pre-peak cyclic shear loading (Thongprapha et al., 2020 [27]) with higher normal stress, the second-order asperities were diminished; subsequently, joint roughness and shear resistance were decreased. Belem et al., 2007 [28] and Belem et al., 2009 [29] investigated joint surface degradation under CNL and CNS for monotonic and cyclic shearing by laser scanning and proposed a method for the estimation of joint surface wearing based on changing normal loads. 

The behaviour of joints is different under CNL and CNS boundary conditions. Elasto-plastic shear stress behaviour was observed on the joints under CNL (Homand-Etienne et al., 1999 [30]; Homand et al., 2001 [31]; Lee et al., 2001 [25]), whereas joints under CNS exhibited strain hardening behaviour (Homand-Etienne et al., 1999 [30]; Homand et al., 200 [31]; Jiang et al., 2004 [32]). 

Few shear strength models have been presented on rock joints under both CNS and cyclic shear loads. Mirzaghorbanali (2013) [33] and Mirzaghorbanali et al. (2013) [34] investigated the shear behaviour of regular rock joints by performing shear tests under CNS with one value of normal stiffness (*k_n_* = 8 kN/mm) induced by a set of springs. Results indicated that with increasing initial normal stress (*P_i_*), the influences of shear rate were less pronounced under the CNS boundary condition. There was no effect of different shear rates on shear strengths with an increase in the number of shear cycles. The authors proposed mathematical shear strength model for regular joints (infilled) under CNS based on initial normal load, initial asperity angle, and the ratio of infill thickness to asperity height. However, cyclic shear behaviour on irregular joints (natural joint roughness) under CNS was investigated by Guansheng et al. (2020) [35]. Rapid decline was observed in shear strength, normal stress, and dilation on the joints for one or two cycles, whereas slow decline was dominant in these parameters after two cycles. They presented an empirical model for shear strength on the joints based on one joint roughness coefficient and three different *k_n_* and 6 cycles.

Rocks with high or medium strength often have roughness or asperities on their joints with the same strength. These joints are not easily subject to shear failure under different boundary conditions. However, soft rock joints or weathered joints are more problematic due to low shear resistance (Richards, 1975 [36]; Barton and Choubey, 1977 [6]; Dearman et al., 1978 [37]; Ozvan et al., 2014 [38]; Ram and Basu, 2019 [39]). Recent works have a high or medium strength of model rock (e.g., 60 MPa) and limitations on *k_n_*, the number of cycles (N), and the asperity angle (i). Hence, in the present study, cyclic shear tests were conducted on simulated soft or weathered rock joints with different regular and irregular asperities under a wide range of *k_n_* (less than, greater than, and equal to the normal stiffness of the joint (*k_nj_*)) to investigate the effect of varying *k_n_* on the shear behaviour of the joints under cyclic loads. Based on experimental results, a shear strength model is also proposed for joints under cyclic shear loads. 

## 2. Approaches to the Simulation of Natural Rock Joint Asperities

**Figure 2:**
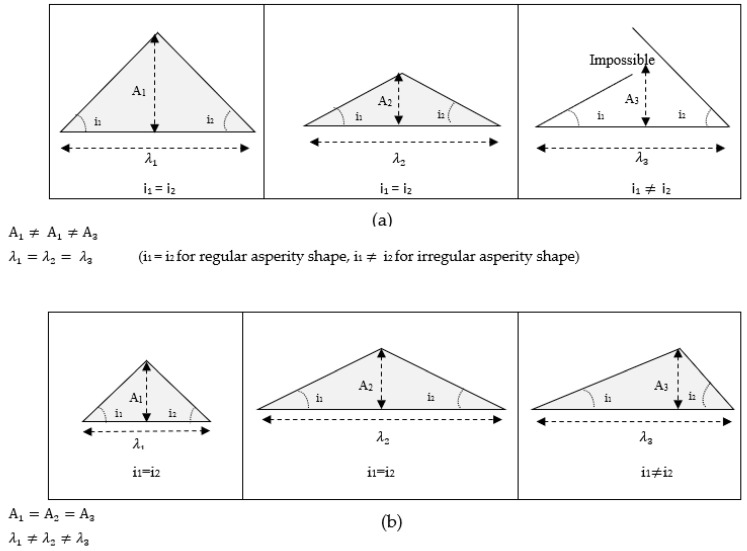
Two different models for joint asperity: (**a**) asperity angle changes based on variation of A, while keeping λ constant, (**b**) asperity angle changes based on variation of λ, while keeping A constant.

Physical modeling of rock joints with regular triangle-shaped asperities has been used in many reports (e.g., Indraratna et al., 1998 [16]; Mirzaghorbanali, 2013 [33], and Zhang et al., 2019 [40]). The change in asperity angle (Figure 2a) is achieved by changing the amplitude of asperity (A) while keeping the base length (λ) constant. We propose to apply a different model where the asperity angle changes by decreasing or increasing the base length, while keeping the amplitude constant. The shape of the asperity can be changed from regular to irregular by changing the lengths and the angles of the two slopes of asperity (Figure 2b). In this study, two regular asperities, 15°-15° and 30°-30°, and one irregular asperity, 15°-30°, were selected to assess the behavior of regular and irregular joints with comparable parameters.

Investigation of natural rock joint profiles (Barton and Choubey, 1977 [6]) shows that the second model is closer to the character of natural rock joints (with increasing joint roughness coefficient, the base length of each asperity is changed or decreased, whereas no significant change was observed in the amplitude (A) of these asperities). In this experimental study, a series of jointed specimens with regular and irregular prismatic shapes of asperities (triangular) was prepared from plaster of Paris as the model material. Joints with asperity 15°-15°, 30°-30°, and 15°-30° were used in the experiment. 

## 3. Experimental Methodology

### 3.1. Experimental Material and Specimen Preparation

In order to simulate soft rock or weathered joints, plaster of Paris was selected as model material, because of its universal availability and because its long-term strength is independent of time once the chemical hydration is completed. The prescribed percentages of water and plaster of Paris were determined so as to obtain the proper workability of the paste and the required strength to simulate the soft rock. The plaster of paris with a water-powder ratio of 0.6 was mixed in a rubber bucket for 2 min and then poured into the special steel moulds with inside dimensions of 299 mm × 299 mm × 85 mm, placed on a vibrating table. An aluminium asperity plate was placed on the bottom of each mould before pouring the paste. The paste in the moulds was vibrated for 30 s to remove the air bubbles from the material. After 60 min, the hardened specimens were removed from the moulds and then left for 20 days at room temperature for air curing. 

**Figure 3:**
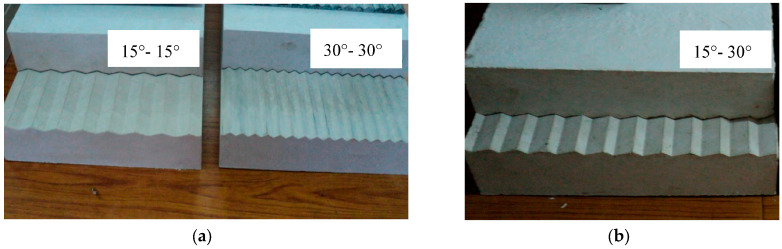
Specimens (**a**) with 15°-15° and 30°-30° as regular asperities and (**b**) 15°-30° as an irregular asperity.

The specimens were formed with joint asperity angles of 15°-15° and 30°-30° as regular joints, and 15°-30° as irregular joints by using different asperity plates (Table 1 and Figure 3). The hardened plaster, having an uniaxial compressive strength of 6 MPa, is a suitable model material for weathered joints or soft rocks like siltstones, claystones, shales, coal, and rock salt. The tangent modulus, Poisson ratio, cohesion, and intact friction angle of the specimens were 2.45 GPa, 0.27, 0.72 MPa, and 51.3°, respectively.

### 3.2. Automatic Shear Testing Machine

**Figure 4:**
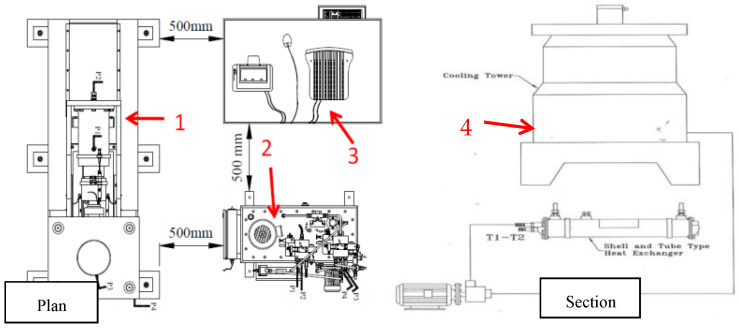
Schematic diagrams of the shear testing machine used for experiments: (1) loading unit, (2) hydraulic power pack with servo valve, (3) controlling unit and data acquisition, (4) cooling unit (water circulation).

An automatic static and cyclic shear testing machine (Niktabar et al., 2018 [41]) under different boundary conditions was used to conduct cyclic shear tests under CNL and CNS with varying normal stiffness. The stiffness was provided by vertical hydraulic pressure based on joint dilation and servo valve feedback programming on the software of the apparatus. The shear testing machine is a combination of four main units, including a frame and loading unit, a hydraulic power pack, a data acquisition and control unit, and a cooling unit as shown in Figure 4. The size of each shear box is 300 mm × 300 mm × 448 mm and the maximum capacities of servo valve normal and horizontal load cells are 500 kN and 1000 kN, respectively. Programming for CNS boundary conditions based on Equation (1) is performed in the software of the apparatus.

(1) $$P=P_{i}+k_{n}\left(∆Y\right)$$

where

*P* = normal stress [MPa]

*P_i_* = initial normal stress [MPa]

*k_n_* = normal stiffness [MPa/mm]

∆*Y* = dilation resisted by the surrounding rock mass [mm]

### 3.3. Testing Procedures 

**Figure 5:**
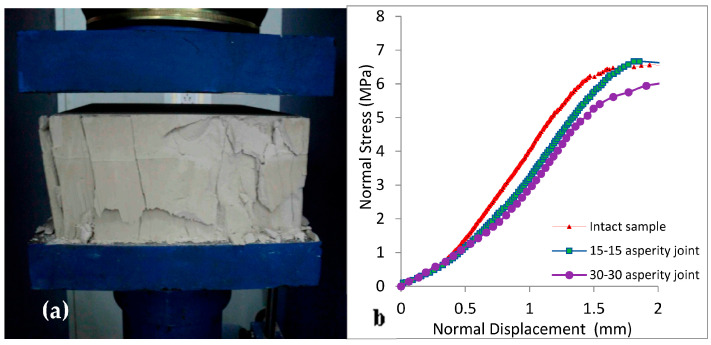
(**a**) Normal stiffness test and (**b**) normal stress vs. normal displacement graph for intact and jointed specimens.

When conducting shear tests under the CNS boundary condition, it is important to apply the relevant normal stiffness value on the shear apparatus to achieve the correct shear resistance on the rock joint. It can be less than, greater than, or equal to *k_nj_* based on the joint at different depths or stiffnesses of the bolts in the reinforced slope in the field. In the present study, uniaxial compressive strength tests were performed on intact and jointed specimens to obtain *k_nj_*, as shown in Figure 5. In order to obtain only the stiffness of the joints, the stiffness of the jointed specimens was measured and deducted from the stiffness of the intact specimen. The representative value of *k_nj_* was 0.71 MPa/mm (64 kN/mm) for the joints.

Cyclic shear behaviour of the joints was investigated under CNL and CNS boundary conditions with different values of *k_n_*. The applied *k_n_* on the joints varied in the range from 0 to 1.42 MPa/mm. An initial normal stress of 0.1 MPa was applied in the present study. It is considered low stress because, as the initial normal stress with respect to the uniaxial compressive strength of specimens increases, the influence of normal stiffness significantly decreases. For the CNL condition, *k_n_* was kept at zero. The input test parameters are presented in Table 2.

## 4. Results and Discussion

### 4.1. Joint with Regular Asperities (15°-15° and 30°-30°) 

**Figure 6:**
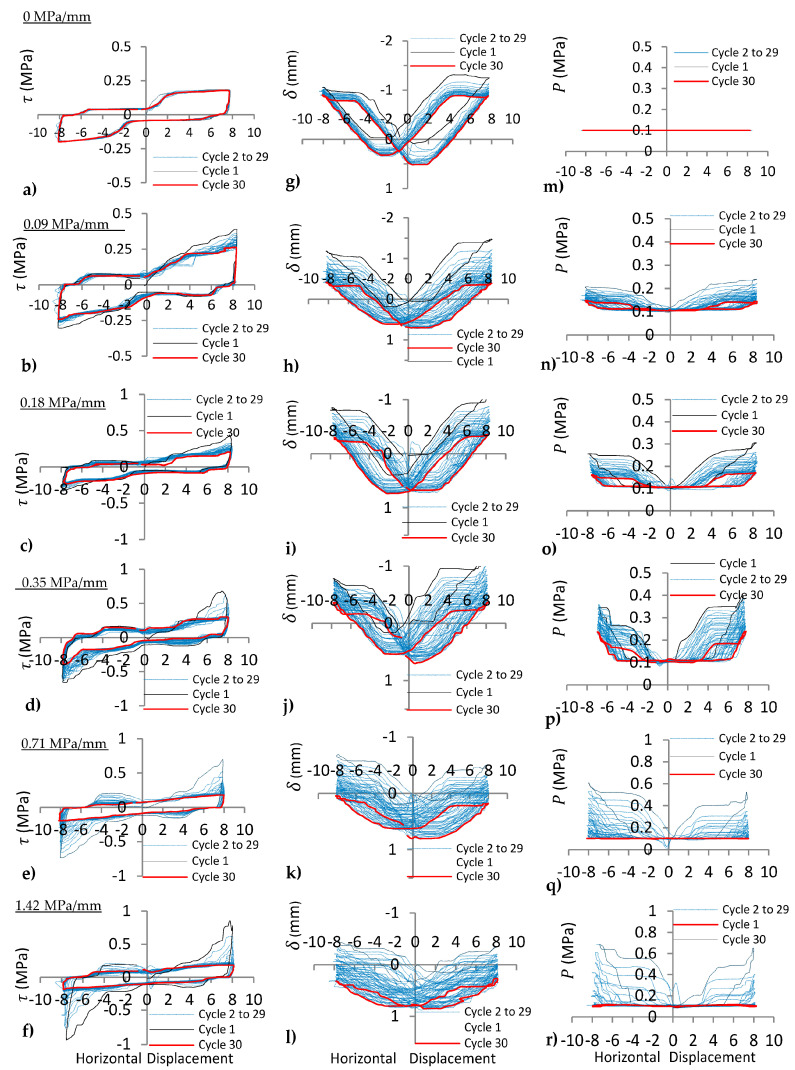
(**a**–**f**) shear stress (*τ*), (**g**–**l**) normal displacement (*δ*), and (**m**–**r**) normal stress (*P*) vs. horizontal displacement of 15°-15° joints under cyclic shear load for *k_n_* = 0, 0.09, 0.18, 0.35, 0.71, 1.42 MPa/mm, respectively.

**Figure 7:**
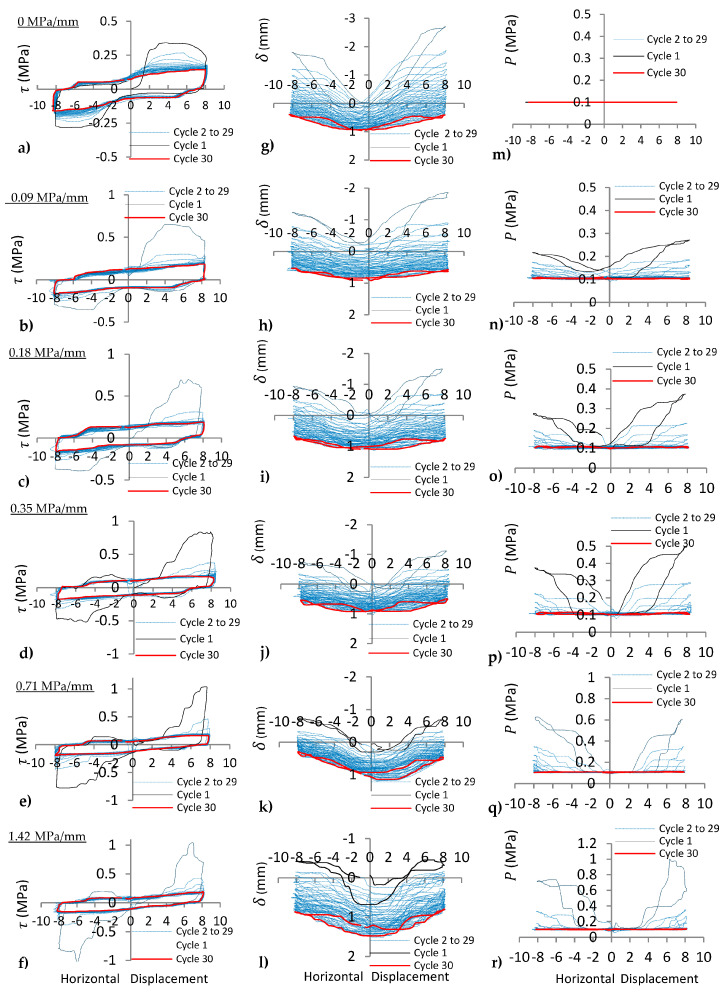
(**a**–**f**) shear stress (*τ*), (**g**–**l**) normal displacement (*δ*), and (**m**–**r**) normal stress (*P*) vs. horizontal displacement of 30°-30° joints under cyclic shear load for *k_n_* = 0, 0.09, 0.18, 0.35, 0.71, 1.42 MPa/mm, respectively.

Experimental results for cyclic shear tests on regular joints under varying *k_n_* (0, 0.09, 0.18, 0.35, 0.71, 1.42 MPa/mm) with the same *P_i_* = 0.1 MPa are illustrated in Figure 6 and Figure 7. The effect of different *k_n_* on dilation, normal stress, and shear stress of the joints can be evaluated as follows.

#### 4.1.1. Shear Stress (*τ*) of the Joints under Varying *k_n_*

Shear stress results indicated that the shear behaviour was sliding on 15°-15° joint asperities (Figure 6a) along the joint for *k_n_* = 0 MPa/mm due to the same peak of shear stress observed from the first to the thirtieth shear cycles. However, peak shear stress decreases on 30°-30° joint asperities (Figure 7a) as the number of cycles increases, i.e., with increasing asperity angle (or joint surface roughness), the shearing mechanism changed from sliding to diminishing of asperities under cyclic loads. 

The effect of increasing normal stiffness (*k_n_*) is manifested by a significant increase in peak shear stress, as indicated in Figure 6a–f and Figure 7a–f. It is due to the increase in normal stress (Figure 6m–r and Figure 7m–r) on the shearing plane at the first cycle. The elasto-plastic shear stress behaviour dominated under CNL or *k_n_* = 0 MPa/mm (Figure 6a and Figure 7a), but strain hardening behaviour (Figure 6b–f and Figure 7b–f) was observed under CNS for all range of *k_n_* (0.09–1.42 MPa/mm).

#### 4.1.2. Normal Displacement (*δ*) of the Joints under Varying *k_n_*

Dilation (negative normal displacement) and dilation angle (slope of normal displacement vs. horizontal displacement curve) of the joint with 15°-15° asperity for *k_n_* = 0 MPa/mm were relatively constant (Figure 6g) and only small subsidence occurred on the joint during thirty shear cycles, i.e., 15°-15° asperities remained intact on the joint. However, both dilation and dilation angle changed for the 30°-30° joint asperity under the same conditions (Figure 7g) because some of the asperities were demolished. With increasing *k_n_* from 0.09 to 1.42 MPa/mm, normal displacement curves moved from dilation (negative normal displacement) to compression (positive normal displacement) as depicted in Figure 6h–l and Figure 7h–l. As *k_n_* increases on the joints, peak dilation decreases, whereas peak normal stress increases for the first cycle. As the number of cycles increases, both peak dilation and peak normal stress decreases because of asperity degradation during cyclic loading.

#### 4.1.3. Normal Stress (*P*) of the Joints under Varying *k_n_*

Normal stress remained constant for *k_n_* = 0 during the tests (Figure 6m and Figure 7m), whereas under CNS (*k_n_* ≠0 MPa/mm), normal stress was a function of dilation. With increasing normal stiffness from 0 to 1.42 MPa/mm, the peak dilation decreases, while peak normal stress increases. This is clear from Figure 6m–r and Figure 7m–r. 

With increasing *k_n_*, the peak normal stress on the joints increases several times more than the initial normal stress (*P_i_* = 0.1 MPa) for the first cycle (see n–r in Figure 6 and Figure 7), but decreases as the number of shear cycle increases. The peak normal stress of the 30°-30° joint (Figure 7n–r) was greater than the peak normal stress of the 15°-15° joint (Figure 6n–r), due to the higher angle of slope (greater joint dilation). However, it decreases to *P_i_* after few cycles, because joint degradation was significant for the 30°-30° asperity.

### 4.2. Joint with Irregular Asperity (15°-30°)

**Figure 8:**
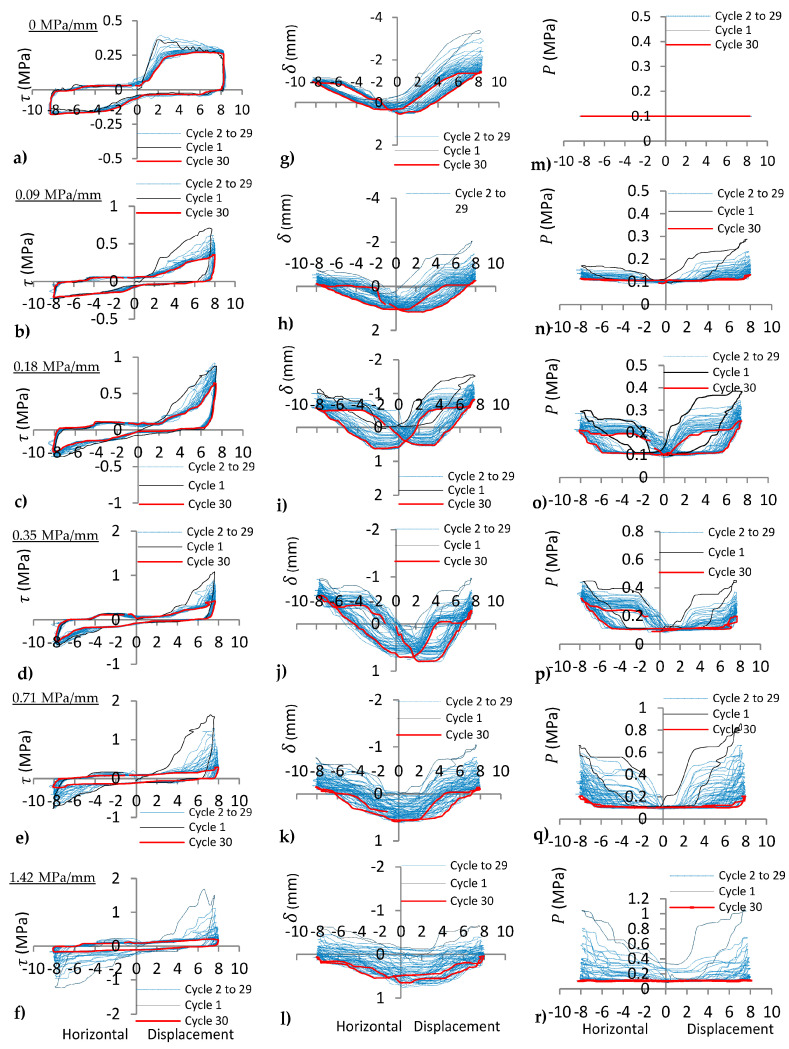
(**a**–**f**) shear stress (*τ*), (**g**–**l**) normal displacement (*δ*), and (**m**–**r**) normal stress (*P*) vs. horizontal displacement of 15°-30° joints under cyclic shear load for *k_n_* = 0, 0.09, 0.18, 0.35, 0.71, 1.42 MPa /mm, respectively.

Experimental results on the joints with irregular asperity (15°-30°) under varying normal stiffness with the same *P_i_* (0.1 MPa) are illustrated in Figure 8. The peak shear stress reduction was greater for the 30° slope of asperity than the reduction for the 15° slope under cyclic movement (Figure 8a–f). The shear behaviour of the joint after several cyclic shear loads under CNS with *k_n_* = 0.71 MPa/mm and *k_n_* = 1.42 MPa/mm changed from non-planar to planar joint behaviour (Figure 8e,f), whereas this behaviour was not observed for *k_n_* = 0, 0.09, 0.18, and 0.35 MPa/mm. This is because complete compression (positive normal displacement) of asperities at low *k_n_* did not take place during shearing. At high *k_n_*, the normal stress during the shearing process increased sufficiently to complete compression of asperities after several cycles; therefore, the planar behaviour was observed on the joint after several cycles. The dilation and normal stress behaviours of the irregular joint were different from the regular joints at low *P_i_* under CNS. Peak dilation (Figure 8g–l) and peak normal stress (Figure 8n–r) were observed on the slope of the asperity with higher angle (30°) under cyclic movement. The behaviour of regular and irregular joints was investigated under CNL for cyclic loads (Niktabar et al., 2017 [42]). The peak shear stress, dilation angle, and asperity diminishing on regular joints between loading and unloading (forward and backward movements) were the same, whereas for irregular joints, they were different. Asperity diminishing was predominant on the slopes of asperities with higher angles on irregular joints until both angles of the asperities became equal after several cycles (Figure 8g). However, in the present study, with increasing normal stiffness (*k_n_* = 0.71 and 1.42 MPa/mm) of the joint, asperity diminishing occurred on both slopes of the irregular joint. This is due to sufficient increasing normal stress on both slopes of the asperity under the CNS condition. Therefore, decreasing peak dilation (Figure 8k,l) and peak shear stress (Figure 8e,f) were observed for both forward and backward shear movements. This experiment indicated that behaviour of the irregular joint was different for CNL and CNS conditions under cyclic loading.

### 4.3. Comparison between the Joints under Varying k_n_

**Figure 9:**
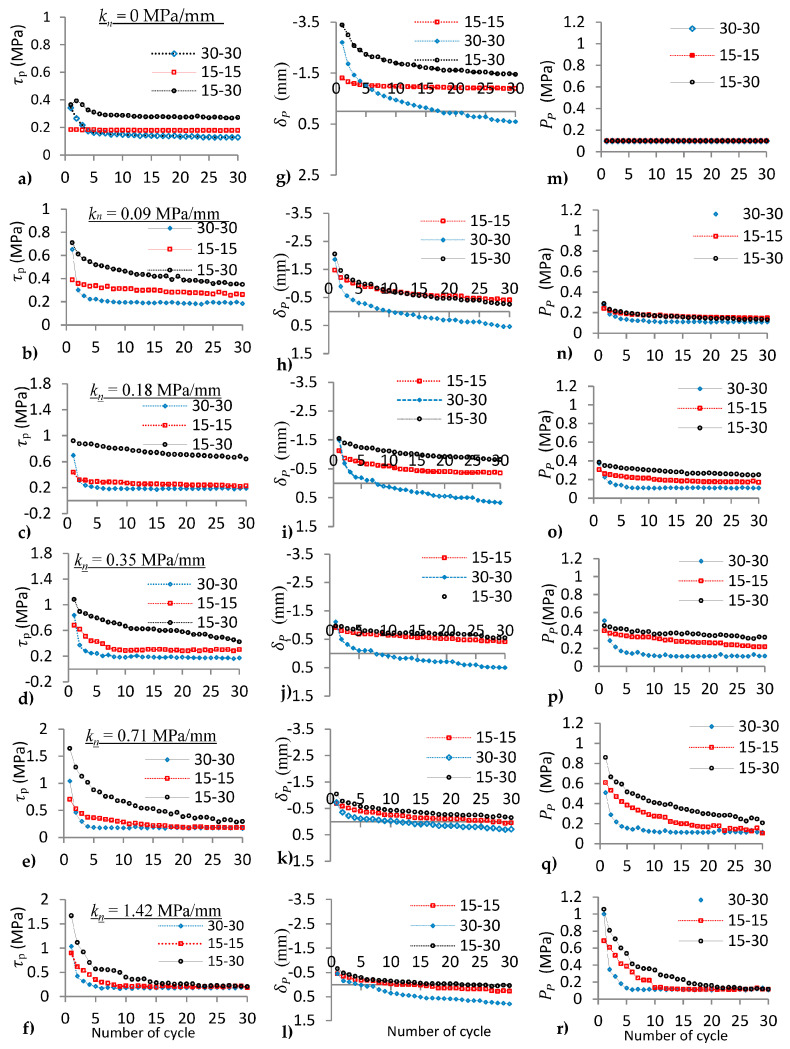
(**a**–**f**) Peak shear stress (*τ*_p_), (**g**–**l**) peak normal displacement ($\delta_{P}$), and (**m**–**r**) peak normal stress ($P_{P}$) vs. horizontal displacement of the joints (15°-15°, 30°-30°, and 15°-30°) under cyclic shear load for *k_n_* = 0, 0.09, 0.18, 0.35, 0.71, 1.42 MPa/mm, respectively.

Peak shear stress, peak dilation (normal displacement), and peak normal stress of the regular and irregular joints (15°-15°, 30°-30°, and 15°-30°) vs. the number of loading cycles were plotted to compare the cyclic behaviour of all the joints under varying normal stiffness (see Figure 9). The first peak shear stress of the joints increases as *k_n_* increases (Figure 9a–f). The peak shear stress on the joint with irregular asperity (i_1_ ≠ i_2_) was observed to be greater than the peak shear stress on the joints with regular asperity (i_1_ = i_2_) in all ranges of normal stiffness and different cycles. 

Peak shear stress on the 30°-30° joint asperity was greater than the peak shear stress on the 15°-15° asperity ($\tau_{P\left(30^{0}\right)}>\tau_{P\left(15^{0}\right)})$ at initial cycles (N < 3) for CNL (*k_n_* = 0 MPa/mm). However, after three cycles (N > 3), the peak shear stress on the 15°-15° joint asperity started to be greater than the peak shear stress on the 30°-30° asperity angle ($\tau_{P\left(30^{0}\right)}<\tau_{P\left(15^{0}\right)})$, as shown in Figure 9a. This is because of different shear mechanisms on the joints. 

Peak shear stress under CNS changed after only one cycle, as shown in Figure 9b, i.e., the peak shear stress of the 15°-15° joint was greater than the peak shear stress of the 30°-30° joint after the first cycle. Hence, the reduction in peak shear stress under CNS was more than that of CNL for cyclic loads, as compared in Figure 9a,b. This is because normal stress under CNS increases significantly at the initial cycles; therefore, joint degradation increases, and subsequently peak shear stress decreases significantly on the joint, whereas a joint under CNL has constant normal stress on the joint, hence joint degradation and peak shear stress decrease gradually under the CNL condition. Degradation of the rough joint (30°-30°) was observed at fewer shear cycles under CNS, when compared with CNL.

Changing peak shear stress of the 15°-15° joint was different from the 30°-30° joint, as *k_n_* increases under cyclic loads (Figure 9). Peak shear stress (shear strength) on the 15°-15° joint was constant for *k_n_* = 0 MPa/mm during thirty cyclic loads (Figure 9a), then it was linearly decreased for *k_n_* = 0.09 MPa/mm (Figure 9b). It decreased sharply, i.e., showing exponential decay, at high normal stiffness (*k_n_* = 0.35, 0.71, 1.42 MPa/mm), as shown in Figure 9d–f. Exponential decay dominated on the peak shear stress of 30°-30° joints for all ranges of normal stiffness (from *k_n_* = 0 to *k_n_* = 1.42 MPa/mm) under cyclic loads. Changing peak shear stress of both joints was similar for high normal stiffness (Figure 9e,f) during cyclic loads.

The difference in peak shear stress between the first and second cycles increased with increasing *k_n_* on the joints (see Figure 9 and Table 3). This difference was 8%, 52%, and 14% for the lowest *k_n_* (0.09 MPa/mm) and 24%, 60%, and 33% for the highest *k_n_* (1.42 MPa/mm) under CNS on three different joints (15°-15°, 30°-30°, and 15°-30°), respectively, as presented in Table 3. Hence, the greatest difference in peak shear stress was observed between the first and second cycles on the joint with higher *k_n_* and asperity angle (30°-30°), i.e., as the joint roughness and *k_n_* increased, degradation of asperities was significant at the first cycle because of greater normal stress on the joint. Therefore, peak shear stress was dramatically decreased for the second cycle.

Peak normal stress and peak shear stress on the joints were investigated under CNS with varying *k_n_* at the first cycle. An increase in peak normal stress with respect to *P_i_* at different *k_n_* was calculated as a percentage and is presented in Table 4. Similarly, an increase in peak shear stress at different *k_n_* with respect to peak shear stress at *k_n_* = 0 MPa/mm as a percentage is presented in the same table. With increasing normal stiffness under the CNS boundary condition, peak normal stress increases at the first cycle. The first peak normal stress of the 30°-30° joint was increased by 280% and 900% with respect to *P_i_* = 0.1 MPa for *k_n_* = 0.18 MPa/mm and *k_n_* = 1.42 MPa/mm, respectively (Table 4). The influence of CNS was not observed after several cycles due to the absence of dilation on the joint. By comparison of the joints at *k_n_* = 1.42 MPa/mm, peak normal stress was increased 900 % for the 30°-30° joint and 570% for the 15°-15° joint, i.e., increasing normal stress under CNS was more effective on the joint with higher asperity angle at the first cycle.

**Figure 10:**
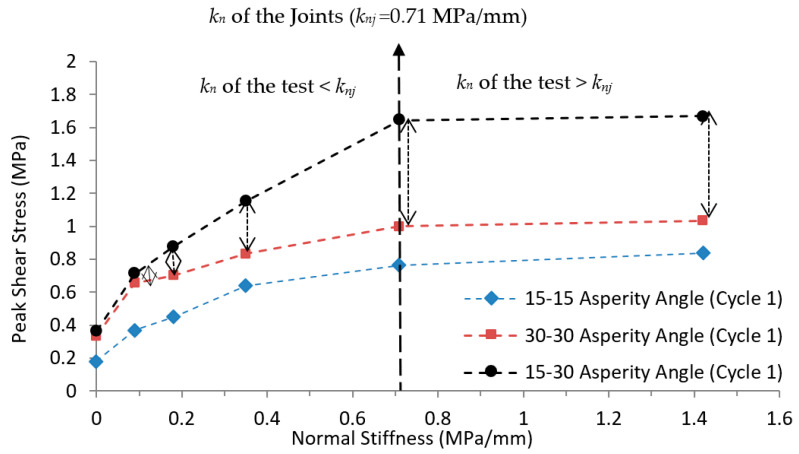
Peak shear stress vs. normal stiffness for regular and irregular joints with different asperity angles at *P_i_* = 0.1 MPa for the first movement.

Peak shear stress for regular and irregular joints at different *k_n_* for the first cycle were compared, as presented in Figure 10. Results indicated that peak shear stress on the joint with irregular asperity (15°-30°) was greater than peak shear stress on the regular joint (30°-30°) under varying *k_n_* for the first movement. The differences in peak shear stress between the regular and irregular joints were 8.2%, 8.3%, 25.0%, 38.4%, 64.3% and 61.3% for *k_n_* = 0, 0.09, 0.18, 0.35, 0.71, 1.42 MPa/mm, respectively. Peak shear stress of the joints increased as *k_n_* increased until it reached *k_nj_*. After that, the effect of increasing *k_n_* declined and no significant change was observed for peak shear stress. The difference in peak shear stress between regular and irregular joints increases with increasing *k_n_* of the test. The maximum difference in peak shear stress between these two types of joints (30°-30° and 15°-30°) was observed at 64.3% on *k_nj_*. However, the minimum difference was observed at 8.2% under CNL. No significant change was observed beyond *k_nj_*.

## 5. Prediction of Shear Strength under Cyclic Loads

**Figure 11:**
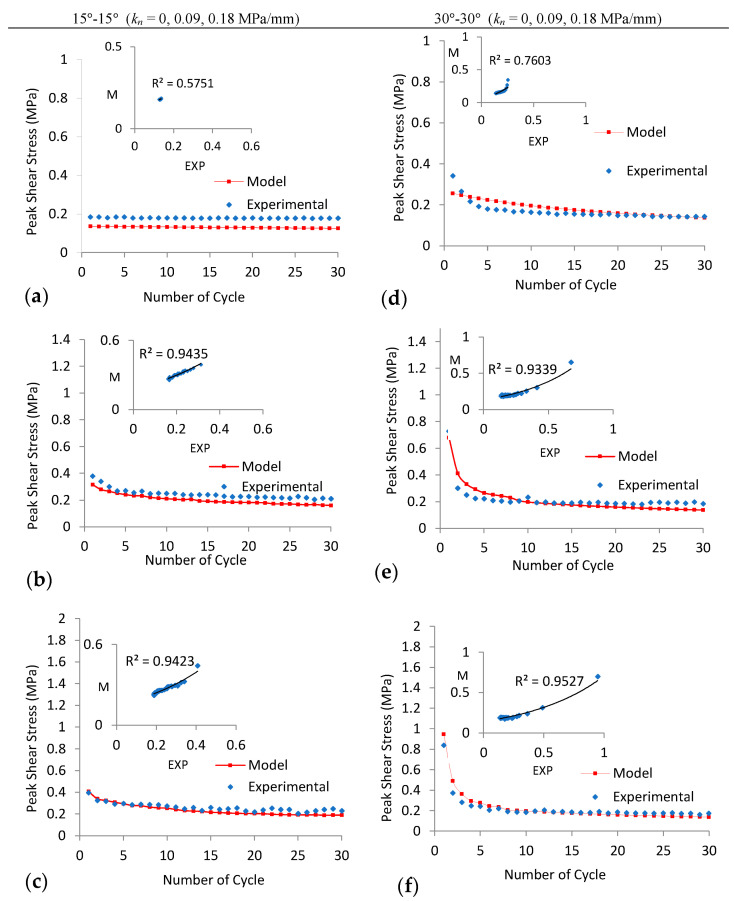
Peak shear stress for both the model (M) and experimental (EXP) results for 30 cycles ((**a**–**c**) 15°-15° and (**d**–**f**) 30°-30°) at *P_i_* = 0.1 MPa under CNS with *k_n_* = 0, 0.09, 0.18 MPa/mm, respectively.

**Figure 12:**
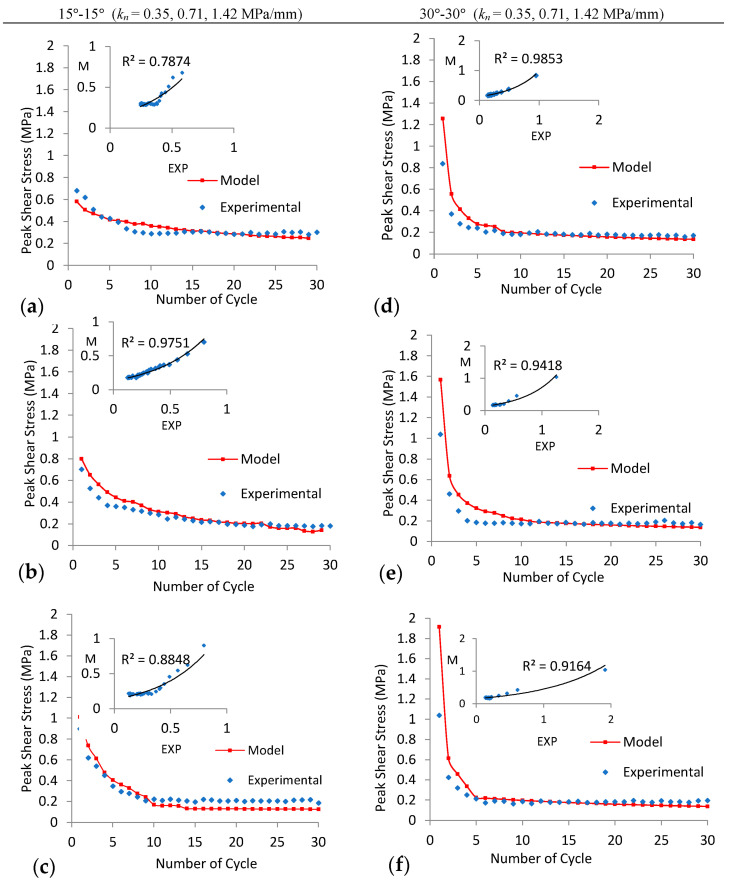
Peak shear stress for both the model (M) and experimental (EXP) results for 30 cycles ((**a**–**c**) 15°-15° and (**d**–**f**) 30°-30°) at *P_i_* = 0.1 MPa under CNS with *k_n_* = 0.35, 0.71, 1.42 MPa/mm, respectively.

Peak shear stress on the joints decreases as the number of cycles increases for all experiments (except for 15°-15° joint under CNL with *P_i_* = 0.1 MPa), as shown in Figure 9. This is due to the asperity angle decreasing exponentially during shear cycles, as described by Plesha (1987), Homand et al. (2001), and Lee et al. (2001). Based on the exponential decay in the asperity angle with the increasing number of cycles and Patton (1966) criteria, a new model (Equations (2)–(4)) is developed here to predict the peak shear stress of regular joints under CNL and CNS conditions for cyclic loads. The proposed model was compared with experimental results for different joint asperities (15°-15° and 30°-30°) under varying normal stiffness for thirty cycles, as illustrated in Figure 11 and Figure 12. A good correlation (exponential regression) was observed between the proposed model and experimental results under varying *k_n_* (only the correlation coefficient is low for the 15°-15° joint under CNL, due to sliding of the joint without diminishing of asperities (Figure 11a)).

(2) $$\tau_{P_{N}}=P_{p}\tan\left(\phi_{b}+i\prime \right)$$

(3) $$i\prime =i\times e^{-2j_{\alpha }\left[4\left(N-1\right)\left({\frac{\left(P_{p}\right)}{\sigma_{c}}}^{2}\right)\right]}$$

(4) $$j_{\alpha }=\frac{i}{\lambda }$$

where

$\tau_{P_{N}}$ = peak shear stress for cycle N [MPa]

*i* = asperity angle [°]

λ = asperity base length [mm]

$j_{\alpha }$ = interlocking factor

i′ = asperity angle after N shear cycles [°]

$P_{p}$ = peak normal stress [MPa]

$\sigma_{c}$ = uniaxial compressive strength of intact specimen [MPa]

*N* = number of cycles

$\phi_{b}$ = basic friction angle [°]

The number of cycles (N) and peak normal stress ($P_{p})$ can change in the model under cyclic loads. $P_{p}$ for different $k_{n}$ is calculated using Equation (1) with insert in: $P_{i}$, *k_n_* and $\delta_{P}\;$(peak dilation) but other parameters are constant in the prediction model during shear cycles. $P_{p}=P_{i}$ for *k_n_* = 0 (CNL). The basic friction angle is 38.7° for the joint. The compressive strength ($\sigma_{c}$) of the specimens is 6 MPa, and peak dilation ($\delta_{P}$) can be obtained from a normal displacement graph (absolute peak dilations are listed for different cycles in Table 5 and Table 6; if the joint was under compression, dilation is considered as zero). The model is presented for a regular joint, but it can be used for irregular joints as well. In case of irregular joint, it may underpredict the shear strength.

The validity of the equations can be obtained by putting the upper and lower boundaries as the number of cycles. If we put N = 1 as the lower boundary in Equation (3), it will create a static or monotonic condition which is the same as the Patton model (1966). Similarly, if we put N = infinity in Equation (3) as the upper boundary, then *i*′ = 0 and it will create the case of a planar joint. Similar observations have also been made during experimental investigation.

## 6. Conclusions

In this study, cyclic shear tests were conducted on simulated soft (or weathered) rock joints with regular and irregular asperities under a wide range of normal stiffness (less than, greater than, and equal to *k_nj_*) to investigate behaviour of the joints under dynamic loads.

(1) Increase in the first peak shear stress was observed as the normal stiffness increases up to the normal stiffness of the joints (*k_nj_* = 0.71 MPa/mm). Beyond the normal stiffness of the joints, no significant change was observed in peak shear stress, i.e., practically inducing normal stiffness on the joints by the bolts or other reinforcements is only applicable until *k_nj_*.

(2) Peak shear stress on irregular joints was greater than on regular joints for all ranges of normal stiffness during cyclic loads. The difference in peak shear stress between regular and irregular joints increases as the normal stiffness increases. The minimum difference of peak shear stress was observed (8.2%) under CNL and the maximum difference was found (64.3%) on normal stiffness of the joint under CNS. These results could provide useful insight in practical rock mechanics and geomaterials studies using the shear strength model of a rock joint.

(3) The difference in peak shear stress between the first and subsequent cycles increases as the joint roughness and normal stiffness increases. It is important when dynamic loads like earthquakes occur on a rock mass or the joints, because shear strength significantly decreases after the first loading cycle.

(4) A new shear strength model is developed to predict peak shear stress of soft (or weathered) rock joints based on varying normal stiffness and different asperity angles under cyclic shear loads.

(5) This study can be extended by conducting cycle shear tests on bolted jointed rock with different normal stiffnesses to investigate the behaviour of reinforced jointed rock under dynamic loads.

## Figures and Tables

**Table 1:** Joint asperity parameters of the specimens.

Joint Type	Asperity Angles i_1_,i_2_ [°]	Asperity Base Length λ [mm]	Asperity Amplitude A [mm]
Regular	15°- 15°	37.3	5
Regular	30°- 30°	17.3	5
Irregular	15°- 30°	27.3	5

**Table 2:** Cyclic shear test parameters.

Parameters	Values
Normal stiffness, *k_n_* [MPa/mm]	0, 0.09, 0.18, 0.35, 0.71, 1.42
Initial normal stress, *P_i_* [MPa]	0.1
Frequency [Hz]	0.01
Amplitude of shear load [mm]	±8
Number of cycles, *N*	30

**Table 3:** Difference in peak shear stress between the first and the second cycles for the lowest and highest *k_n_* under CNS.

*k_n_* = 0.09 MPa/mm				*k_n_* = 1.42 MPa/mm		
Joint Asperity i_1_,i_2_ [°]	First Peak Shear Stress [MPa]	Second Peak Shear Stress [MPa]	Difference (%)	First Peak Shear Stress [MPa]	Second Peak Shear Stress [MPa]	Difference (%)
15°-15°	0.39	0.36	8	0.70	0.53	24
30°-30°	0.61	0.30	52	1.04	0.42	60
15°-30°	0.71	0.61	14	1.67	1.12	33

**Table 4:** First peak normal stress and shear stress related to increasing normal stiffness on regular and irregular joints for *P_i_* = 0.1 MPa.

*k_n_* [MPa/mm]	*P_i_* [MPa]	Joint Asperity i_1_,i_2_ [°]	Peak Normal Stress *P_p_* [MPa]	Increase in *P_p_* with Respect to *P_i_* [%]	Peak Shear Stress $\tau_{p}$ [MPa]	Increase in $\tau_{p}$ with Respect to CNL [%]
0 (CNL)	0.1	30-30	0.10	0	0.34	0
0.09	0.1	30-30	0.27	170	0.65	91
0.18	0.1	30-30	0.38	280	0.70	106
0.35	0.1	30-30	0.51	410	0.83	144
0.71	0.1	30-30	0.62	520	1.00	194
1.42	0.1	30-30	1.00	900	1.00	194
0 (CNL)	0.1	15-15	0.10	0	0.18	0
0.09	0.1	15-15	0.24	140	0.39	117
0.18	0.1	15-15	0.3	200	0.45	150
0.35	0.1	15-15	0.40	300	0.65	261
0.71	0.1	15-15	0.59	490	0.76	322
1.42	0.1	15-15	0.67	570	0.84	367
0 (CNL)	0.1	15-30	0.10	0	0.36	0
0.09	0.1	15-30	0.29	190	0.71	97
0.18	0.1	15-30	0.38	280	0.89	147
0.35	0.1	15-30	0.45	350	1.08	200
0.71	0.1	15-30	0.86	760	1.64	356
1.42	0.1	15-30	1.05	905	1.67	364

**Table 5:** Peak dilations on the joint with 30°-30° asperity under different normal stiffness during 30 cycles for *P_i_* = 0.1 MPa (if there was no dilation or only compression, then peak dilation is 0 or $\delta_{P}=0$).

N	0.09 [MPa/mm]	0.18 [MPa/mm]	0.35 [MPa/mm]	0.71 [MPa/mm]	1.42 [MPa/mm]
1	1.86	1.50	1.11	0.72	0.46
2	0.90	0.69	0.50	0.35	0.16
3	0.56	0.39	0.32	0.22	0.11
4	0.41	0.21	0.19	0.15	0.05
5	0.30	0.19	0.11	0.11	0.00
6	0.28	0.10	0.10	0.09	0.00
7	0.28	0.10	0.00	0.09	0.00
8	0.11	0.00	0.00	0.06	0.00
9	0.06	0.00	0.00	0.03	0.00
10	0.01	0.00	0.00	0.02	0.00
11	0.00	0.00	0.00	0.01	0.00
12	0.00	0.00	0.00	0.00	0.00
13	0.00	0.00	0.00	0.00	0.00
14	0.00	0.00	0.00	0.00	0.00
15	0.00	0.00	0.00	0.00	0.00
16	0.00	0.00	0.00	0.00	0.00
17	0.00	0.00	0.00	0.00	0.00
18	0.00	0.00	0.00	0.00	0.00
19	0.00	0.00	0.00	0.00	0.00
20	0.00	0.00	0.00	0.00	0.00
21	0.00	0.00	0.00	0.00	0.00
22	0.00	0.00	0.00	0.00	0.00
23	0.00	0.00	0.00	0.00	0.00
24	0.00	0.00	0.00	0.00	0.00
25	0.00	0.00	0.00	0.00	0.00
26	0.00	0.00	0.00	0.00	0.00
27	0.00	0.00	0.00	0.00	0.00
28	0.00	0.00	0.00	0.00	0.00
29	0.00	0.00	0.00	0.00	0.00
30	0.00	0.00	0.00	0.00	0.00

**Table 6:** Peak dilations on the joint with 15°-15° asperity under different normal stiffness during 30 cycles for *P_i_* = 0.1 MPa, (if there was no dilation or only compression, then peak dilation is 0 or $\delta_{P}=0$ ).

N	0.09 [MPa/mm]	0.18 [MPa/mm]	0.35 [MPa/mm]	0.71 [MPa/mm]	1.42 [MPa/mm]
1	1.38	1.12	0.91	0.68	0.45
2	1.18	0.87	0.80	0.59	0.35
3	1.12	0.82	0.77	0.51	0.29
4	1.02	0.77	0.74	0.44	0.21
5	0.94	0.72	0.69	0.39	0.17
6	0.89	0.67	0.69	0.36	0.19
7	0.89	0.67	0.69	0.36	0.19
8	0.81	0.62	0.65	0.32	0.09
9	0.76	0.60	0.69	0.26	0.07
10	0.73	0.60	0.64	0.24	0.02
11	0.71	0.54	0.64	0.23	0.00
12	0.69	0.49	0.63	0.22	0.02
13	0.73	0.48	0.59	0.18	0.01
14	0.62	0.47	0.59	0.23	0.00
15	0.60	0.44	0.56	0.14	0.00
16	0.58	0.43	0.58	0.14	0.00
17	0.57	0.41	0.56	0.12	0.00
18	0.56	0.41	0.54	0.11	0.00
19	0.56	0.41	0.52	0.10	0.00
20	0.56	0.41	0.52	0.10	0.00
21	0.56	0.41	0.52	0.10	0.00
22	0.55	0.39	0.49	0.11	0.00
23	0.47	0.37	0.47	0.04	0.00
24	0.47	0.37	0.47	0.04	0.00
25	0.47	0.37	0.47	0.04	0.00
26	0.45	0.38	0.45	0.05	0.00
27	0.42	0.38	0.44	0.00	0.00
28	0.45	0.36	0.45	0.00	0.00
29	0.41	0.38	0.43	0.00	0.00
30	0.41	0.36	0.41	0.00	0.00

## Data Availability

Some or all data, models, or code generated or used in this study are available from the corresponding author by request.
